# Acceptability of Mental Health Apps for Aboriginal and Torres Strait Islander Australians: A Qualitative Study

**DOI:** 10.2196/jmir.5314

**Published:** 2016-03-11

**Authors:** Josie Povey, Patj Patj Janama Robert Mills, Kylie Maree Dingwall, Anne Lowell, Judy Singer, Darlene Rotumah, James Bennett-Levy, Tricia Nagel

**Affiliations:** ^1^ Darwin Remote Mental Health Service Top End Mental Health Service Northern Territory Department of Health Darwin Australia; ^2^ Menzies School of Health Research Institute of Advanced Studies Charles Darwin University Casuarina Australia; ^3^ Menzies School of Health Research Institute of Advanced Studies Charles Darwin University Alice Springs Australia; ^4^ School of Health Charles Darwin University Darwin Australia; ^5^ University Centre for Rural Health (North Coast) University of Sydney Lismore Australia

**Keywords:** mobile apps, mental health, indigenous populations, therapeutics, cognitive behavioral therapy, acceptance and commitment therapy, culturally competent care

## Abstract

**Background:**

Aboriginal and Torres Strait Islander Australians experience high rates of mental illness and psychological distress compared to their non-Indigenous counterparts. E-mental health tools offer an opportunity for accessible, effective, and acceptable treatment. The AIMhi Stay Strong app and the ibobbly suicide prevention app are treatment tools designed to combat the disproportionately high levels of mental illness and stress experienced within the Aboriginal and Torres Strait Islander community.

**Objective:**

This study aimed to explore Aboriginal and Torres Strait Islander community members’ experiences of using two culturally responsive e-mental health apps and identify factors that influence the acceptability of these approaches.

**Methods:**

Using qualitative methods aligned with a phenomenological approach, we explored the acceptability of two culturally responsive e-mental health apps through a series of three 3-hour focus groups with nine Aboriginal and Torres Strait Islander community members. Thematic analysis was conducted and coresearcher and member checking were used to verify findings.

**Results:**

Findings suggest strong support for the concept of e-mental health apps and optimism for their potential. Factors that influenced acceptability related to three key themes: personal factors (eg, motivation, severity and awareness of illness, technological competence, and literacy and language differences), environmental factors (eg, community awareness, stigma, and availability of support), and app characteristics (eg, ease of use, content, graphics, access, and security and information sharing). Specific adaptations, such as local production, culturally relevant content and graphics, a purposeful journey, clear navigation, meaningful language, options to assist people with language differences, offline use, and password protection may aid uptake.

**Conclusions:**

When designed to meet the needs of Aboriginal and Torres Strait Islander Australians, e-mental health tools add an important element to public health approaches for improving the well-being of Aboriginal and Torres Strait Islander people.

##  Introduction

### Aboriginal and Torres Strait Islander Mental Health and Service Access

Aboriginal and Torres Strait Islander Australians experience much higher rates of psychological distress compared to non-Indigenous Australians [1]. Tragically, consequences such as suicide also occur at higher rates—twice that of non-Indigenous Australians [2]. Younger Aboriginal and Torres Strait Islander Australians are at the greatest risk, with suicide rates 5.9 (female) and 4.4 (male) times higher than non-Indigenous Australians aged 15 to 19 years [3]. Despite this, 35% of Aboriginal and Torres Strait Islander people with high to very high levels of psychological distress report difficulties accessing health services [1]. Barriers include ineffective communication, differences in worldview from Western treatment models, stigma, and distance to appropriate services [4].

Treatment approaches that equalize power and facilitate genuine communication are favored [5]. Two-way learning, incorporating local knowledge and worldview into treatment, is most likely to be effective [6-8]. Access to such services in rural and remote areas is limited and technological innovation provides an important opportunity to bridge geographic and sociocultural divides.

### The Potential of E-Mental Health

E-mental health approaches use electronic media for the delivery of therapy/treatment [9] and are emerging as a safe, therapeutically effective, and acceptable treatment option for common mental health concerns [10]. They have the potential to increase access by overcoming barriers such as distance and cost, and improve flexibility by being available at times suitable to the person, with relative anonymity if desired [11]. Recognizing these benefits, in 2012 the Australian Government released its National e-Mental Health Strategy including a comprehensive commitment to improving access for all Australians [9].

Some studies have shown Internet-delivered cognitive behavioral therapy (CBT) to be as effective as face-to-face therapy for depression, anxiety, and social phobia [10,12,13]. However, these findings cannot be generalized to all Australians. Many online CBT treatments recommend 4 to 6 hours of weekly participation and require medium to high literacy levels, regular access to the Internet, and a high level of computer and Internet competence. Given these criteria, it is not surprising that many studies report variable adherence and completion rates [14,15] raising questions about satisfaction with e-mental health approaches [16]. Understanding what drives acceptability is an important next step for successful e-mental health uptake.

### Technology in the Aboriginal and Torres Strait Islander Health Context

Despite the potential appeal of e-mental health approaches, the vast majority of effectiveness, accessibility, and acceptability findings to date relate to non-Indigenous Australians. Aboriginal and Torres Strait Islander people generally have less access to technology than non-Indigenous Australians [17]. Significant investment by the Australian Government is being made to increase access through programs such as the National Broadband Network [9]. Increased access has led to increased use. Estimates suggest 60% to 80% of people 10 years and older living in some remote Northern Territory (NT) communities have access to and use a mobile phone regularly [18]. In these settings, mobile phones are mostly used for communication with family and Internet browsing; however, they have also been shown to support health care treatment and clinical trial retention [18,19].

Locally produced eHealth strategies that use culturally appropriate graphics and videos, limited text, and Aboriginal and Torres Strait Islander voices reportedly have the best chance of successful implementation [20,21].

### Introducing Two Culturally Responsive E-Mental Health Tools: AIMhi and ibobbly

Two culturally responsive e-mental health tools have recently been developed with local input: the AIMhi Stay Strong iPad app and the ibobbly suicide prevention app [22,23].

The Australian Integrated Mental Health Initiative (AIMhi) began in the NT in 2003 and sought to incorporate local Aboriginal and Torres Strait Islander knowledge and worldviews into treatment. Extensive community consultation resulted in the generation of a culturally responsive brief therapy (AIMhi Stay Strong Plan). This brief therapy was tested in a randomized controlled trial (RCT) in 2009. Results showed significant improvements in well-being, substance use, and self-management following the therapy [24]. The AIMhi Stay Strong Plan is a therapist-supported strengths-based brief intervention integrating motivational interviewing and low-intensity CBT techniques [25]. It follows a 4-step process exploring family, strengths, and worries, before goal setting. The goal-setting phase integrates the strengths and worries discussion into specific lifestyle changes chosen by the client and adapted to their values and sociocultural context [24].

**Figure 1:**
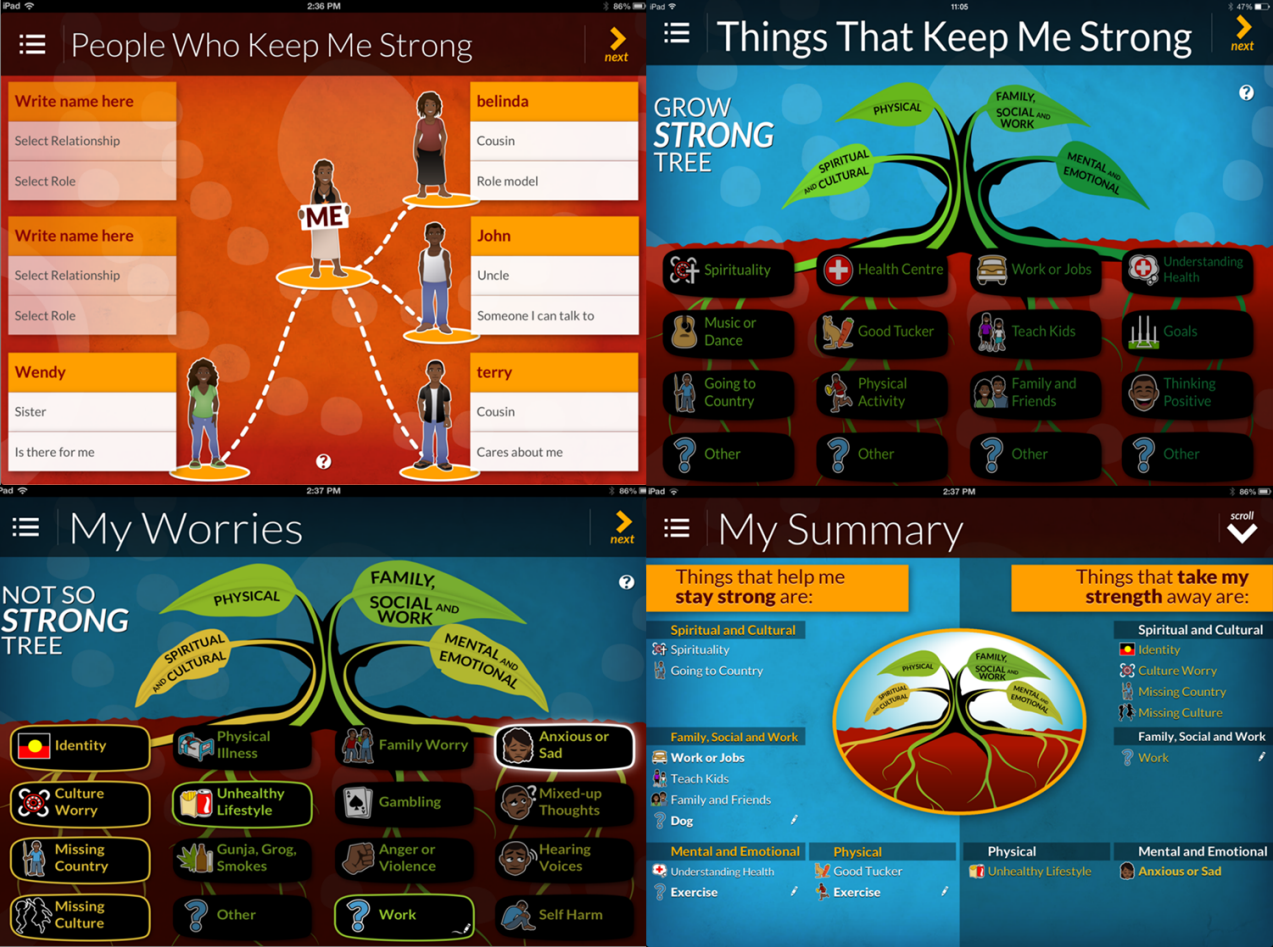
Screenshots of AIMhi app. Reproduced with permission.

The AIMhi Stay Strong app (Figure 1) translated the paper-based care plan into an electronic format. The app uses colorful graphics, audio, and animation with limited text. Information is entered through selecting icons, typing text, and drop-down selection boxes, with an option of including a photograph of the client. Care plans can be saved, stored, copied, emailed, printed, and reaccessed to facilitate an ongoing therapeutic monitoring tool. Once downloaded, the app does not require continuous Internet connection for use.

**Figure 2:**
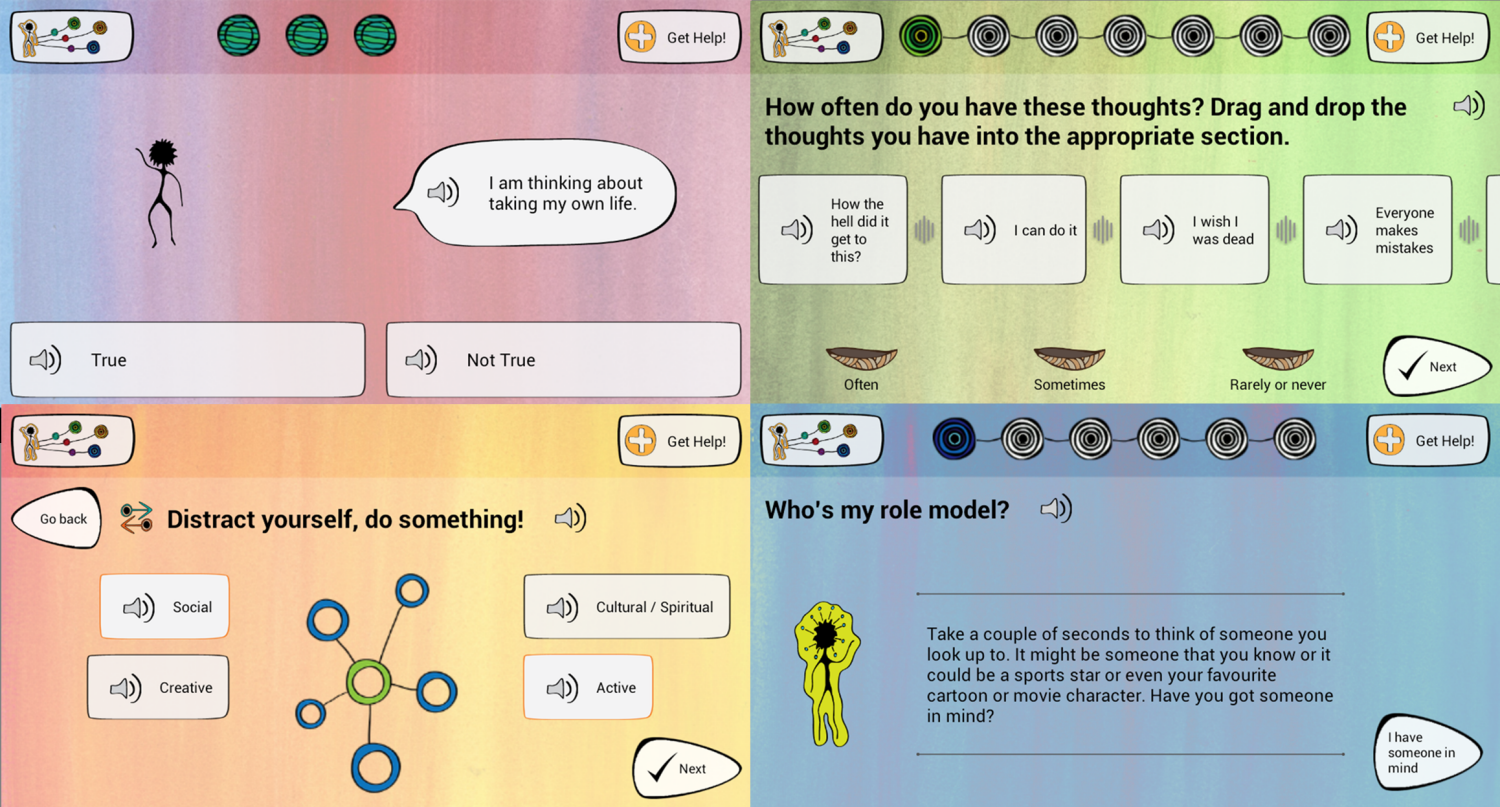
Screenshots of ibobbly app. Reproduced with permission.

The ibobbly suicide prevention app (Figure 2) was developed in northern Western Australia (WA) for Aboriginal people aged 18 to 35 years. The app is based on acceptance and commitment therapy and uses mindfulness and values-based action strategies. The app is a self-help tool and includes three self-assessment and three activity modules. Self-assessment modules ask the user if they are experiencing intrusive thoughts, including thoughts of suicide; if so, they are directed to call 000 Emergency, Lifeline, or Kidshelp line. Three activity modules use interactive activities, stories, and videos, aiming to help users manage upsetting thoughts and emotions, identify ideals, and set small, realistic goals. An audio icon on every page assists people with limited literacy. A summary page prompts reflection. Once downloaded, the app does not require continuous Internet connection.

The first version of ibobbly was evaluated through a pilot study in the Kimberley region, WA. In all, 61 participants completed the trial. Preliminary analysis suggests that the group using ibobbly had reductions in psychological distress, depression, and suicidal thinking (personal written communication, F Shand, May 27, 2015). Results will be published soon. A national RCT began in 2015.

The Australian Government’s e-Mental Health Strategy seeks to increase availability of e-mental health services and provide training and support to primary, allied health and Aboriginal and Torres Strait Islander Health Workers through the e-Mental Health in Practice (eMHPrac) Project [9]. The present study is linked with the eMHPrac project. E-mental health services may provide an opportunity to deliver structured, cost-effective, and accessible mental health services to Aboriginal and Torres Strait Islander people; however, their acceptability is not well understood. This study aimed to explore Aboriginal and Torres Strait Islander community members’ experiences of using two culturally responsive e-mental health apps and identify factors that influence the acceptability of these approaches.

## Methods

We used a qualitative design, aligned with a phenomenological approach, to explore the experiences and perspectives of participants in relation to the apps. A series of three focus groups were held with participants who identified as members of the Aboriginal and Torres Strait Islander community in Darwin, NT. The design drew from a larger study conducted in northern New South Wales [26]. The design, sampling, and recruitment strategy were discussed with the Aboriginal researcher and an expert reference group that guides other e-mental health projects in the NT to ensure local relevance. Ethics approval was granted through the Human Research and Ethics Committee for the NT Department of Health and Menzies School of Health Research, Darwin, NT including Aboriginal and Torres Strait Islander subcommittee.

### Recruitment

Purposive sampling was used, aiming to recruit 6 to 8 Aboriginal and Torres Strait Islander community members. Local service providers (education, health, and community) were asked to provide nominations based on inclusion/exclusion criteria. Eligible participants were aged at least 18 years, able to attend all three 3-hour groups, willing and capable of talking in a group setting in English, did not have a florid or severe level of mental illness, identified as Aboriginal or Torres Strait Islander, had basic familiarity with computers, and were not currently employed as a health worker. Prospective participants provided demographic information and addressed the preceding criteria through an expression of interest form. The research team selected participants for maximum variation, aiming for even numbers of male/females and a wide range of ages.

### Data Collection

Three focus groups were held in a period of one week in December 2014. The groups were facilitated by a female non-Indigenous researcher and a male Aboriginal researcher. Both facilitators have experience working with Aboriginal and Torres Strait Islander people with mental illness, one as a remote public service clinician and one as a mentor and e-mental health cotrainer. Each focus group started with a statement requesting confidentiality from group members, risk management strategies, a short introductory video, and a brief demonstration of how to use each app. Participants were then asked to use the apps individually (ibobbly) or in pairs (AIMhi). The difference in process reflected the different developer recommendations; the AIMhi app is a therapist-guided intervention, whereas the ibobbly app is designed as a self-driven tool. The sessions then reviewed participants’ experiences of using the apps and factors they thought may influence acceptability. Participants were reimbursed for their time, transport, and other expenses by an AUS $80 shopping voucher per 3-hour session. Written informed consent for participation and voice recording was obtained from all research participants. The sessions were voice recorded and back-up field notes were taken, including researcher reflections and observations.

### Data Analysis

The first author (JP) led the analysis in consultation with the Aboriginal researcher (PPJRM). Audio recordings were transcribed by the first author and data from all sources (transcripts, field notes) were entered into NVivo qualitative data analysis software version 10 (QSR International Pty Ltd). Initial inductive analysis identified emerging themes which were further refined through collaborative analysis between the first author and the Aboriginal researcher. This strengthened the authenticity of findings because the analysis process was informed by an Aboriginal perspective. A thematic map was developed and discussed within the research team. A member-checking group was run 5 months following the initial focus groups, involving three members of the initial groups. These members were selected because they varied in age and gender, showed interest and enthusiasm, and were available. Reimbursement was equivalent to the initial focus groups. The aim of the member-checking session was to review the thematic map and main findings with participants, who confirmed that these reflected their experiences and perspectives related to acceptability of the apps. It is likely data saturation was achieved given that the methodology chosen resulted in a dataset that was both rich (detailed and nuanced commentary) and thick (several hours of interactive discussion). Triangulation of data sources, coresearcher checking, and member-checking strategies were used to enhance the trustworthiness of the findings.

## Results

### Participants

Ten expressions of interest were received through four service providers. A total of nine people (3 male, 6 female) were accepted into the focus groups. One female was excluded to ensure more equal gender distribution. Ages ranged from 18 to 60 years with a mean age of 33 (SD 17) years. All participants identified as Aboriginal or Torres Strait Islander, resided in the local area, and identified English as the main language they spoke at home. Eight of 9 (88%) participants provided an email address, 7 of 9 (77%) identified they had access to the Internet at home, and 2 of 9 (22%) owned a mobile phone with Internet capability. Eight of 9 (88%) identified they were interested in the topic.

Nine participants attended the first focus group (100%), 8 attended the second (88%), and 6 attended the third (66%). The Aboriginal researcher was only able to attend the first group and member-checking group. Reasons given for nonattendance included funeral attendance, family, and work commitments.

### Overview and Thematic Map

All participants expressed enthusiasm and optimism for the concept of an app and the progressiveness of improving mental health and well-being using apps:

I like the app idea; I think it is fantastic, I think it is great to move with the times.

56-year-old female

Specific benefits identified were the opportunity to reach larger audiences, the ability to provide immediate access to help, ability for an individual to have greater independence, and the possibility of anonymity:

Just being able to intervene in a timely manner and help someone through their difficult struggle.

60-year-old female

I think for the individual it might be an increased level of independence and privacy. You know they can look at the app without having to speak to someone.

50-year-old female

**Figure 3:**
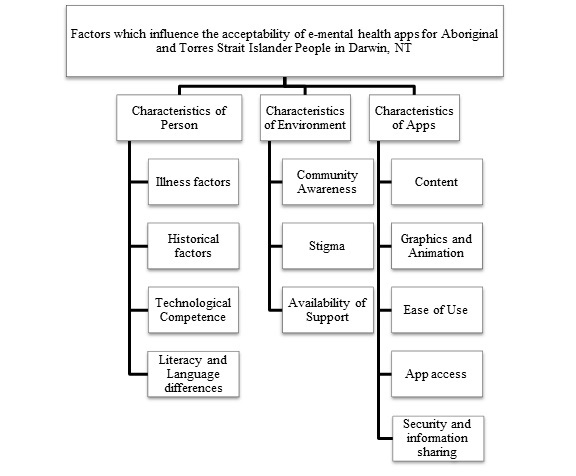
Factors affecting acceptability of e-mental health apps for Aboriginal and Torres Strait Islander people in Darwin, NT.

Three main themes emerged related to the acceptability of e-mental health tools: factors related to the person, the environment, and the apps (see Figure 3).

### Characteristics of Person

Illness factors, including a person’s awareness of their mental illness and motivation to change, were suggested as potential influences on help-seeking and app use:

...if they did have a mental illness, and they were not really aware of it, there would be no reason for them to use the app.

60-year-old female

It is just a matter of the motivation and like wanting to get better and everything.

18-year-old female

Both apps were considered to be most appropriate for people with less severe mental illnesses:

So it could be a good thing for people who have some minor mental health things to help them get along. If you have that there, like Stay Strong, that might help them or encourage them to overcome it. You know-that don’t have really serious mental health issues

AIMhi, 50-year-old female

Historical factors were seen to cause mental ill health and influence acceptability of the apps to participants. The negative impact of colonization on the well-being of Aboriginal and Torres Strait Islander people was highlighted in the discussion, along with uncertainty about the role of apps in addressing such concerns:

I am someone who can sit here and say, I have got a problem, with all that, and there are people who just do whatever to try and get out of it, because they can’t talk about it. I wanna go back to country, I want my song, I want my dance, I want my ceremony, I want my country, they can’t talk about it, and how does it happen. How does this help you deal with those type of things?

AIMhi, 56-year-old female

Two participants expressed a sense of helplessness in preventing suicide. This impacted on their perception of the utility of the apps:

Suicide though, suicide is a very different thing, to other things, it’s very different isn’t it, a state of mind, you know what questions could you ask someone who is in that state of mind.

AIMhi, 56-year-old female

You know it is a big claim calling it a suicide prevention app, maybe another name. Another term for that, I don’t think it is really going to prevent suicide; it is just a tool that someone can use, to go through their emotions and self-assess where they are at.

ibobbly, 60-year-old female

Technological competence was identified as influencing the acceptability of e-mental health apps. One older participant noted:

You know you got some young people who said, yeah it’s pretty easy to use, and they are very more computer literate, and some older people that find it difficult to go back, it is across the board but you need to look at the app [AIMhi] to be user friendly I suppose.

50-year-old female

Literacy and language differences were identified by some participants as potential barriers to app use. Some participants stated that these tools may not be appropriate for their family in remote communities:

My people are from Alice Springs. So yeah, if it is like mob [Aboriginal people] that can’t understand English language, they won’t understand anything, you will have to teach them like pretty much everything I got taught at school.

19-year-old male

### Characteristics of Environment

Community awareness of available e-mental health tools was reported to be very limited. Inclusion in the research increased participant’s awareness:

I—on Wednesday—had no idea this existed. I did have a look on the website and seen a bit and thought, oh yeah that must be it.

AIMhi, 50-year-old female

Suggested strategies to overcome a lack of community awareness included promotion in schools, advertising in newspapers, local radio, television ads, and promotion in health centers by health professionals.

Community involvement in development was identified as important, with some participants questioning potential uptake in other communities:

I think it is great that it is the community who have pushed for the tool to be created; it will be interesting to see how it maps out in that community...I think there is a real need for it, especially that way [Northern WA] and here in the NT...And if it is successful in that community because the community made it, it would be interesting to see, you know sharing it with other areas.

ibobbly, 30-year-old female

Stigma was seen as a barrier for people accessing help:

Well we have to get that stigma out of the way. We have got to deal with that because that is one of the biggest problems and it is a very serious problem.

60-year-old female

One participant noted that apps have the potential to “get around shame job” (26-year-old male). 

Availability of support was identified as an influence on uptake and effective use of e-mental health apps. Participants identified that the clinician-supported nature of the AIMhi app would mitigate some challenges related to motivation, literacy, and familiarity with technology. When asked if you need to know how to use an iPad to use the AIMhi app, one participant noted:

Not if you are working with a clinician. If you are doing it on your own you would have to have knowledge of how to use it.

60-year-old female

Suggestions for other ways of integrating apps into care pathways included their use as a screening tool, communication aid, immediate help option, self-help tool, or in conjunction with face-to-face help.

The need to link the apps to other supports, particularly emergency help, was also identified. The following are comments on the help box on the ibobbly app:

I think it is good that it is there.

18-year-old female

Yeah if someone has access to a phone, they can talk to someone. But yeah, it’s not human, the question, it’s not like someone is actually asking it. If someone was actually asking it, it would be more meaningful.

19-year-old male

Online videoconferencing, instant messenger, and websites were suggested as alternatives and participants felt these could provide a personal touch that may improve outcomes. Participants discussed potential constraints regarding availability of these services due to credit, Internet, or phone access, and suggested any recommended services needed to be free to access from mobile phones.

### Characteristics of Apps

Ease of use was identified as a main factor in facilitating engagement with the apps. Participants who found the apps hard to navigate were less likely to use the app or recommend it to others:

Yeah I don’t know, this is my opinion and everyone is entitled to their opinion, but I don’t see it working—no. Not for any age group. But that is just my thing. I got totally lost, totally confused.

ibobbly, 56-year-old female

Recommendations included clear navigation buttons, a home page or “dashboard” which is easily understood, and the use of checklists or clearly marked progress bars to indicate progress through the apps.

Content gaps discussed (in one or other of the apps) included colonization, intergenerational trauma, identity, methamphetamines, cyberbullying, and the influence of peers. Participants recommended additions to the apps to prompt consideration of these topics.

Participants also identified the need for the apps to have a clear and purposeful journey, where individuals were virtually supported through a journey or story which was relevant to them and ended with resolution:

I thought it would take me on a journey, but I didn’t see that at all. You know what is your problem, how can we help, what can you do, but I didn’t feel that

ibobbly, 56-year-old female

Participants recommended approaches that allowed clients to define their own problems and solutions:

If you word it so people feel comfortable, so I am going to put that in, but only if they are invited, only if they want to say more about themselves, not preaching, not dictating...

56-year-old female

Clear, concise, and relevant language were acknowledged as important. Words that could be difficult to understand needed to be supported by explanations or short video clips, as discussed in the following example in reference to the word “resilience” within ibobbly:

I only really in the last couple of years, found out what resilience means...I sorta relate it to, when I am working and like stressed out; I say my resilience is low, whereas when I am going with the flow I am very resilient. Is that what it means?...Maybe if you have a breakdown of someone giving an example of a particular word.

30-year-old female

The inclusion of Aboriginal and Torres Strait Islander languages was considered to enhance engagement and understanding:

Some people might want to use their own language instead of saying “deadly.” It would be ideally, pie-in-the-sky dreaming, that your language comes up, or you write it in, instead of “deadly” like “manymak” or “gumul.”

ibobbly, 56-year-old female

Graphics and animation were perceived as supporting motivation. Culturally relevant graphics, voices, animation, and optional short video clips may assist in engagement with the content, improve understanding, and overcome literacy issues. Recognizing the diversity of Aboriginal and Torres Strait Islander communities, participants identified the need for regionally specific graphics or language to be described including meanings and interpretations to aid wider acceptability.

Some participants were concerned the metaphors could be interpreted differently by people with low literacy or had concern about the degree to which the metaphors aligned with user interests (eg, only related to males when a football analogy was used to introduce goal setting in the AIMhi app). Modifications suggested included personalization of the graphics and metaphors to enhance relatability.

App access was deemed to be improved by availability, not only on tablet devices, but also on all brands of mobile phones. Cost was perceived to negatively influence access and could be addressed through free download of e-mental health apps for individuals and the option of offline use once downloaded to preserve credit.

Security and information sharing was not discussed by participants until prompted by the facilitators. There was some concern expressed about storage of personal health information on the Internet. However, most participants noted that other personal information being seen would concern them more than information on either app:

Wouldn’t bother me at all, photos and messages and emails and things would bother me more.

60-year-old female

Password protection and the ability to share app information with health professionals and personal electronic heath records were also considered important. One participant suggested that the collection of statistics for service planning would be a logical inclusion in any e-mental health tool:

Well I think that is. Not names, just information. I think it is necessary isn’t it. Isn’t that the whole idea?

60-year-old female

## Discussion

### Principal Results

This is one of the first studies to explore the factors that influence acceptability of e-mental health apps for Aboriginal and Torres Strait Islander community members. This study identifies characteristics of person, environment, and e-mental health apps that influence acceptability. Although no other technology acceptance models currently exist which focus on e-mental health or are tailored to an Indigenous population, our findings have similarities to the Health Information Technology Acceptance Model [27]. This model identifies similar attributes of person, community, and technology (e-tool) as influences on the acceptability and uptake of health information technology.

Personal characteristics reported by our participants to influence acceptability include illness factors (severity of illness, motivation to change, awareness of mental illness), historical factors, technological competence, and literacy and language differences. Our participants’ perceptions that e-mental health approaches are more acceptable for people with less severe mental illnesses are consistent with a similar belief that is widely held by health professionals and community members alike [16,22]. However, others have found e-mental health treatment may also be acceptable for people with more severe longstanding illnesses than often assumed [28]. Therefore, further research is warranted to examine e-mental health tool acceptability for Aboriginal and Torres Strait Islander people with more severe illnesses.

The possession of the skills necessary to make full use of e-mental health tools affects their uptake and adherence [29]. In addition, limited instruction, technological issues, inexperience with mobile Internet browsing, and a lack of motivation have been reasons identified for discontinuation [14]. In line with these findings, participants in this study reported motivation, technological competence, and literacy and language differences as likely to influence acceptability. Participants perceived that apps have broad applicability to people of all ages and varied skill groups, provided they were suited to the target audience. For some Aboriginal and Torres Strait Islander people, English is not spoken at home and English literacy may not be attained through formal education [30]. Aboriginal and Torres Strait Islander people may also have less access to technology than non-Indigenous Australians [17]; therefore, specific app adaptations responding to the needs of the target group will aid uptake.

Within our study, environmental characteristics perceived to impact uptake included community awareness, stigma, and availability of support. The ability to successfully navigate the apps was a direct influence on our participants’ perception of the content, perceived journey, and overall experience. Importantly, when support was given either by the facilitators or other group members, participants’ experiences improved. This offers insight into the potential role friends and family could play in supporting e-mental health tool use in a community setting. Participants suggested that clinician support, which includes Aboriginal Health Workers and primary health care workers, may overcome barriers related to limited English literacy, limited ability with technology, and/or motivation. Other studies have found that introduction of e-mental health tools in a structured environment (eg, schools) or with therapist support, enhance adherence [29,31]. The intention of e-mental health tools is not to replace face-to-face services, rather to complement or offer alternative treatment options for people wanting to access mental health care [9]. Our findings support this goal with participants identifying a clear preference for e-mental health apps to integrate with established treatment pathways rather than as stand-alone interventions.

Our participants identified community involvement in development as a good strategy for improving acceptability, adherence, and uptake of e-mental health apps in a location specific community. This accords with the findings of others and strengthens the evidence for collaborative development of eHealth tools [20,21]. Recognizing the diversity of Aboriginal and Torres Strait Islander communities, our participants identified the need for regionally specific graphics or language to be described, including meanings and interpretations to improve acceptability in other parts of Australia. Such adaptations are particularly relevant given the potentially nationwide availability of e-mental health apps through Internet sharing.

The importance of app design and characteristics should not be underestimated. E-mental health programs need to be attractive to the user and present themselves as a good match to the person’s needs [29]. Others have found that relevant and interesting content and flexible accessibility were reasons identified for initiating and continuing use of e-mental health programs [14]. In keeping with these findings, our participants highlighted the need for apps to be easy to use, contain relevant content and instructions on navigation, have culturally relevant language and graphics, incorporate a clear purposeful journey ending in resolution, include options to support people with language differences, and allow for offline use and password protection. Incorporation of Aboriginal Languages was considered to enhance engagement. Participants highlighted the potential barriers of developing apps in every Aboriginal language and, therefore, suggested alternative ways to incorporate language, such as options for voice recording/playback and text box entry.

Security is considered by many to be “paramount and drive all other considerations” when designing interventions [14,23,32]. In contrast, our participants expressed concern about security only when prompted and felt dissemination of other information stored on their phones (eg, emails, photos) would cause greater concern than information stored on either app. This could partly be attributable to the apps themselves because neither stores personal details (full name, date of birth, contact details). Participants felt that password protection was important, but needed to be balanced with usability because some found the regular inputting of passwords inhibited flow and interest. Others have received similar feedback supporting the need for careful consideration of the balance between security and usability [33].

Our participants considered that lack of compatibility across devices (mobile phone/tablet) and platforms (Apple or Android), high data charges, and limited mobile coverage could be barriers to use. Others have identified similar barriers; however, they suggested that with targeted investment in increasing mobile networks, decreasing usage costs, and increases in technology, these issues may become redundant [14]. Nevertheless, given that rural and remote regions where Aboriginal and Torres Strait Islander people often reside tend to lag behind in terms of such advances [34], such considerations are particularly important in the design of culturally responsive tools.

### Limitations

The size of this study was small with only nine participants drawn from one location. Five of nine participants and the Aboriginal researcher knew one another before being included in the groups. This occurred by chance and did not appear to impact on participant’s willingness to express their views. Nevertheless, the purposive selection process (targeting those with fluency in English and established computer literacy) may have introduced bias resulting in a group more likely to favor the acceptability of these tools.

Three members of the research team were involved in the development of the AIMhi app, including the Aboriginal researcher, thus introducing another potential source of bias toward acceptability. Finally, three of the nine participants were not within the target age range for the ibobbly app (18-35 years) suggesting that comments relating to acceptability of this app need to be interpreted with caution. Despite these limitations, the study allowed active participation by all participants who presented a range of perspectives that have not yet been heard in relation to current innovations in technology and mental health.

### Conclusions

E-mental health apps could be an acceptable way of enhancing services to Aboriginal and Torres Strait Islander people through attention to design that incorporates local community perspectives and thoughtful adaptation according to the target group. Further research with Aboriginal and Torres Strait Islander people to explore effectiveness of e-mental health tools is needed, particularly with a broader target population that recognizes diversity of culture and considers variations in literacy, language, background, and type of well-being concern.

E-mental health tools represent an opportunity to promote mental health awareness, to enhance early intervention strategies, and to promote access to evidence-based treatment. When designed to meet the needs of Aboriginal and Torres Strait Islander people, e-mental health tools add an important element to public health approaches aimed to improve the mental health and well-being of Aboriginal and Torres Strait Islander Australians.

## Abbreviations

AIMhi: Australian Integrated Mental Health Initiative
CBT: cognitive behavioral therapy
eMHPrac: e-mental health in practice
NT: Northern Territory
RCT: randomized controlled trial
WA: Western Australia
